# Acknowledgement to Reviewers of *Insects* in 2013

**DOI:** 10.3390/insects5010270

**Published:** 2014-02-27

**Authors:** Insects Editorial Office

**Affiliations:** MDPI AG, Klybeckstrasse 64, CH-4057 Basel, Switzerland.

The editors of *Insects* would like to express their sincere gratitude to the following reviewers for assessing manuscripts in 2013:
Simone-Findstrom, MichaelShapiro-Iian, DavidRussell, Jacob A.Rodriguez-Saona, CesarWard, DarrenHurst, Mark R. H.Contreras-Garduño, JorgeTragust, SimonRuiu, LucaMedrzycki, PiotrRodriguez, Josephine J.Wraight, S. P.Kühne, StefanRadek, RenateAbd-Alla, AdlyBoykin, Laura M.Williams, CarolineWei, TaiyunYokoyama, Victoria Y.Blouin, NicKariithi, H.M.Uzunboylu, HüseyinJames, RosalindHedge, CloyceVänninen, IreneXu, J.Woodcock, BenGerling, DanBashey, F.Cook, SamRansford, CarolynGriffin, C. T.Avery, Pasco B.Moseley, C.Kaplan, FatmaLima, Juliana ToledoWard, JulianLencioni, ValeriaGrace, KennethJohnson, D.Bowers, M. DeaneCornelius, MaryTypas, Milton A.Poelman, ErikGwiazdowski, RodgerMarcelino, José A. P.Tanaka, ToshiharuAlthouse, Benjamin M.Lopez-Llorca, Luis VicenteDyer, AdrianGluskin, Rebecca T.Pereira, RobertoVan Der Geest, LeoSabater-Munoz, B.Bartonicka, TomasCampos-Herrera, R.Arab, AlbertoToledo, JorgeKadowaki, TatsuhikoOruc, H. H.Papachristos, Dimitrios P.Marchini, DanielaMAINI, StefanoZappala, LuciaBáo, Sônia N.Shutler, DaveConti, BarbaraLino-Neto, JoséPauls, Steffen U.Lewis, VernardViscuso, R.Wiemers, M.Liu, Zhi LongAnfora, GianfrancoCanale, A.Vacas, SandraHannaford, SusannahGries, R.Pekas, ApostolosDietemann, VincentRutledge, Claire E.Oliva, Clelia F.Cunningham, SaulBos, NickLees, RosemarySzeto, W. Y.Dong, KeKilpinen, OleChu, Shu-ChuanShapiro, ArthurDavies, Thomas G. EmyrMarinaki, MagdaleneZehnder, Caralyn B.Anderson, John F.Gaudriault, SophieTorres-Vila, Luis M.Lammers, PeterMantzoukas, SpiridonMoore, DarrellLietze, Verena-UlrikeBishop, A. H.Cruse, HolkGibson, WendyNguyen, Q.Andrew, Richard J.Gonzalez, AngelicaBernal, VicenteLihoreau, MathieuYao, JianxiuDimarco, Romina D.Raine, NigelKim, YonggyunNishida, RitsuoOkutani, F.Hull, JoeHodson, A. K.Biergans, Stephanie D.Oberhauser, KarenPeter Graystock, PeterShvetsov, A. V.Lopez-Ferber, MiguelFeldhaar, HeikeJozet-Alves, ChristelleUnruh, Thomas R.Laing, Mark DelmegeSchmuker, MichaelHoch, GernotSolter, LeellenRyan, KathleenLewis, Leslie C.Qin, ChuanguangHaavik, LaurelTurlings, Ted C. J.Hunter, WayneRigosi, ElisaDuncan, L. W.Jackson, MarkNieh, JamesReilly, KimCuthbertson, Andrew G. S.Ikeno, HidetoshiSoberón, MarioSchumacher, VeronaSpringate, SimonBruns, EmilyLysyk, TimothyFuentealba, AlvaroMullens, BradleyPeterson, JenniferLindgren, StaffanSmilanich, Angela M.Koehler, PhilipRodriguero, MarcelaWang, ChengshuNew, TimothyGross, JürgenVilcinskas, AndreasHaynes, Kenneth F.Thiéry, DenisBulmer, Mark S.Kaufman, ReubenHuang, Rong-nanNorros, VeeraUgelvig, Line V.Mercader, R. J.Gassmann, Aaron J.Larson, Kirk


